# Event and Comment

**Published:** 1910-10-15

**Authors:** 


### DENTAL REGISTER.

**Vol. LXIV.** **October 15, 1910.** **No. 10.**

### EVENT AND COMMENT.

PUBLIC HEALTH. Much time and effort has been given to the discussion of the importance of public sanitation. Pure foods and healthful air and water are the constant factors in every problem that comes up for discussion or action. To these are added of late the question of more sanitary environment. This of course has come about as a consequence of the centralization of the people in large cities where land is scarce and rents are expensive. Most of these problems are being solved, especially in the larger cities where the great need has been so clearly demanded. In the great city of New York, old tenement houses are fast giving away to sanitary dwellings for the poor, and the children in the public schools have ample play grounds under proper surveillance to promote healthful exercise. The subject of medical inspection of children in schools and of workers in factories is finding favor and is being inaugurated in all well meaning communities. All dentists are interested in the propaganda for saving the childrens teeth, especially those who from lack of education and financial ability are not sufficiently interested in these important organs. All this is immensely important and should meet the cordial approval of every dentist who sees more than any one else what great inroads dental caries makes on the most useful organs in the animal economy. Of what use is pure food if it can not be properly masticated or insalivated, or of what use is pure air if it can not be adequately inhaled through its proper channel, the nose?

We have heard much of race suicide from its varying sources, and we always endorse with commendation the "big families;" but, do we ever stop to think that as a race of real producers we should excel not so much by numbers as because of normal capacity? There can be little doubt that a man with a fully developed body with all functional organs in perfect condition will have more power and incentive to achieve than one not so endowed.

Now the point we wish to make in this connection is that the two most important factors in right physical development are the immediate concern of the dentist; and they are a proper development of the bones of the face and the organs contained in them, which have practically everything to do with the healthful nutrition of the body. Man is developed and nourished by what he eats and breathes, and if he can't eat wholesome food or breathe pure air, because of maldevelopment of the teeth or the nose, or because these organs are so diseased that every bit of food or breath of air is contaminated in passing through the mouth or nose how is it possible for him to reach any proper degree of development or maintain a reasonable degree of power and physical health? The dental profession has felt that its duty was done when the losses of disease in the teeth had been artificially repaired in the best way its genius or skill could devise and has left the more important matter of prevention to the medical profession, which has had plenty to occupy its attention in similar efforts of rescue. Is it not now time for both professions to turn their attention more seriously to preventive efforts? It will be necessary to go back to the beginning of life to influence this matter in the most effective way. Mothers should know that the bones of the body and the teeth are beginning to develope at as early a period as the third month of foetal life, and that lack of proper tissue building elements or accidental diseases from that time on are likely to have serious and detrimental influences on the well being of their children. We do not know of any literature on this subject that is accessible even to the profession, and as dentists are seldom consulted on such matters, few dentists have any intelligent idea of what advice they should give to their patients at such times. The writings in our literature on such subjects are so fragmentary and unsatisfactory that we are in no way capable of taking an intelligent attitude even on the subject. There can be no question but we should have some definite knowledge of such matters and we then could hope to influence them in the most effective way. There seems every probability that the present agitation for saving the teeth from decay will help some, and that in the future people will take more pains to keep the teeth free from harmful deposits by an intelligent mouth toilet; but dental caries will still go on in its destructive action and because of mal-occlusion of the teeth and obstructed nasal passages people will suffer with diseases of the gums, and nasal membranes, and be deprived of pure and assimilable food and air. There can be little doubt that much of the present incapacity of children in school work and their restlessness is due to lack of proper nutrition, and the constant vitiation of their normal food elements. This may not be a problem which our profession can work out, but it seems that we could do much to call attention to its bearing on general health problems and thus be the means of starting an agitation that should be fruitful in not only saving the teeth of the race, but establishing a higher standard of general health which is eminently proper and worth while.

